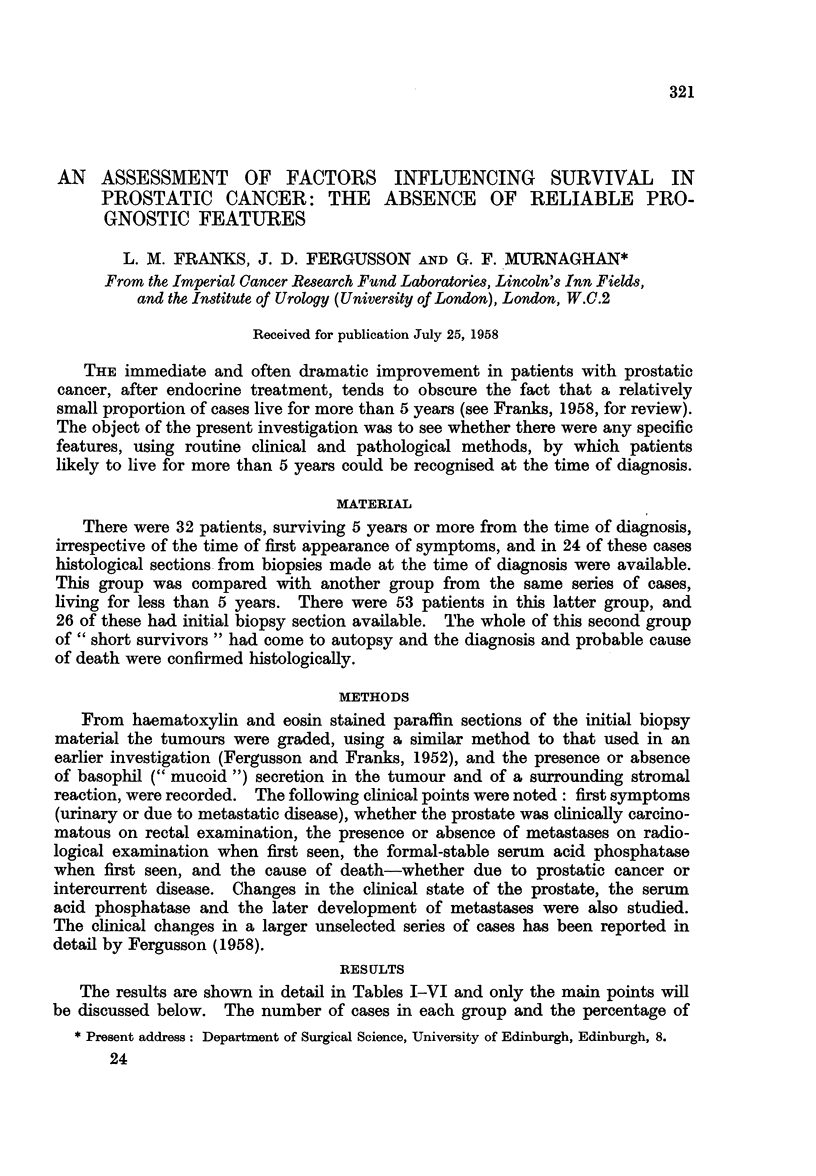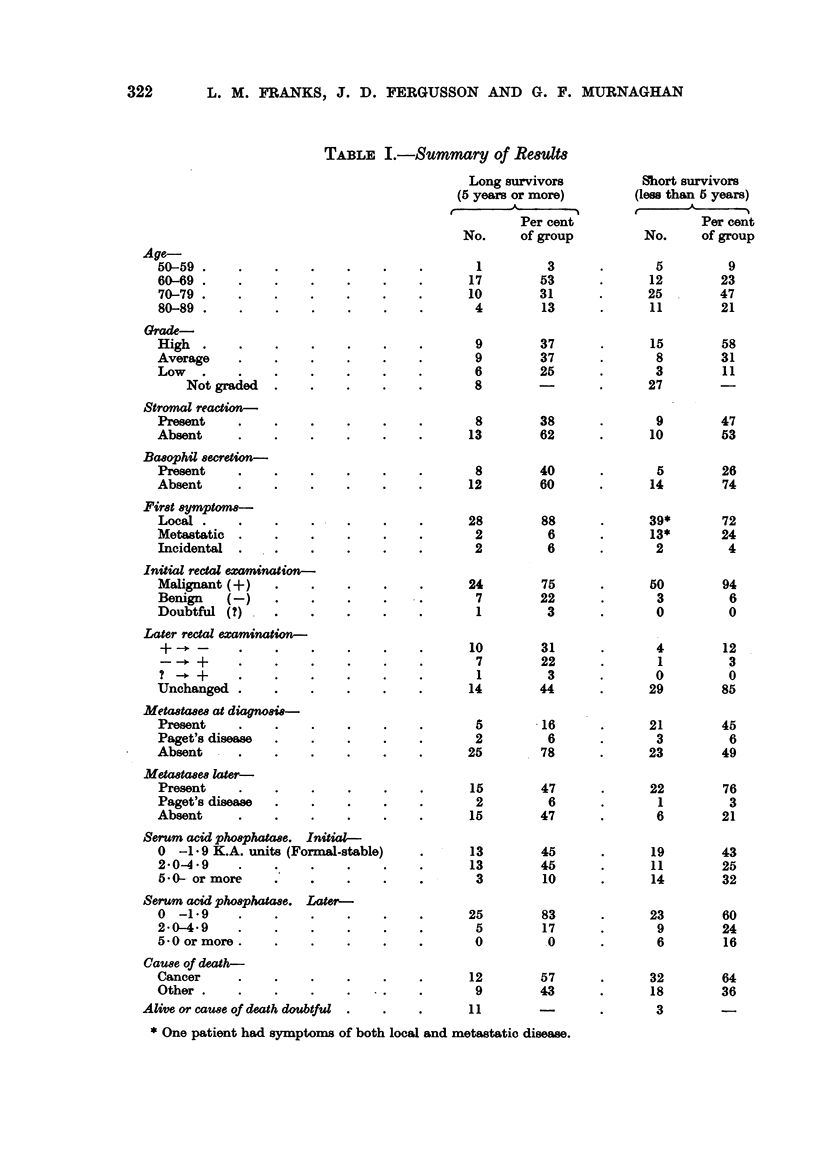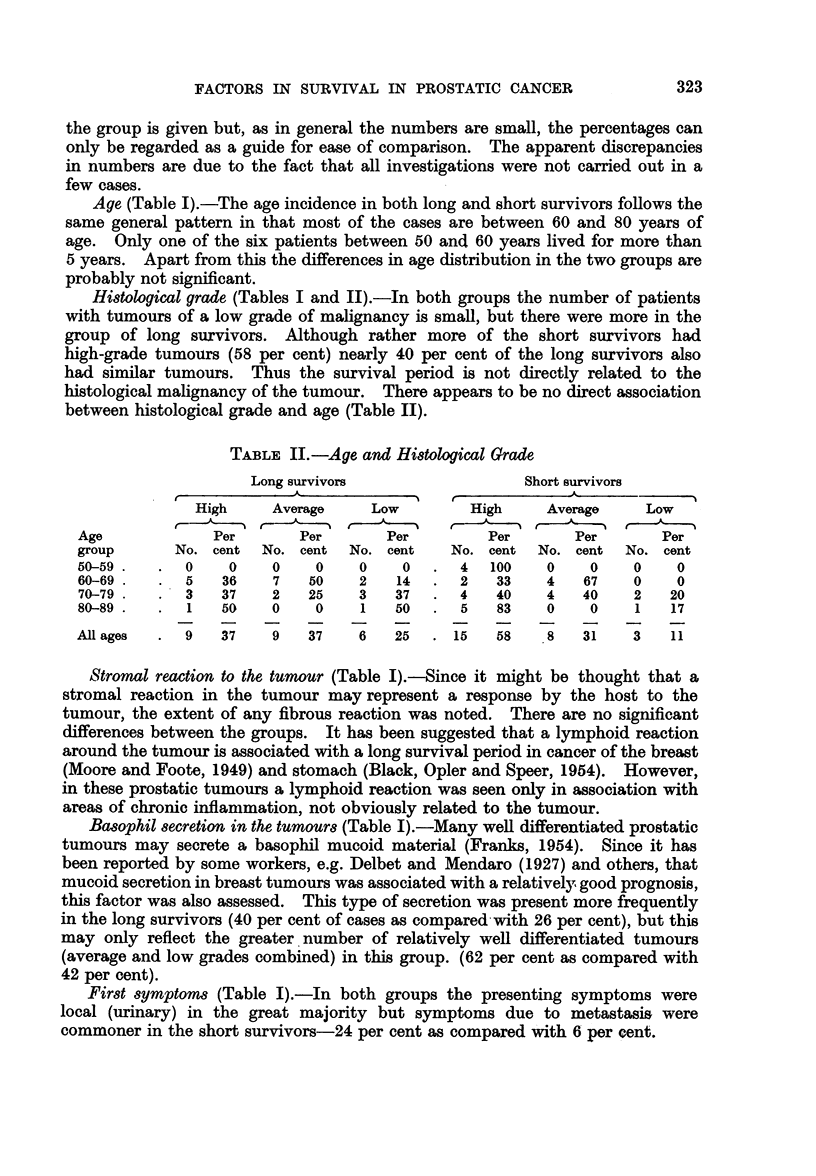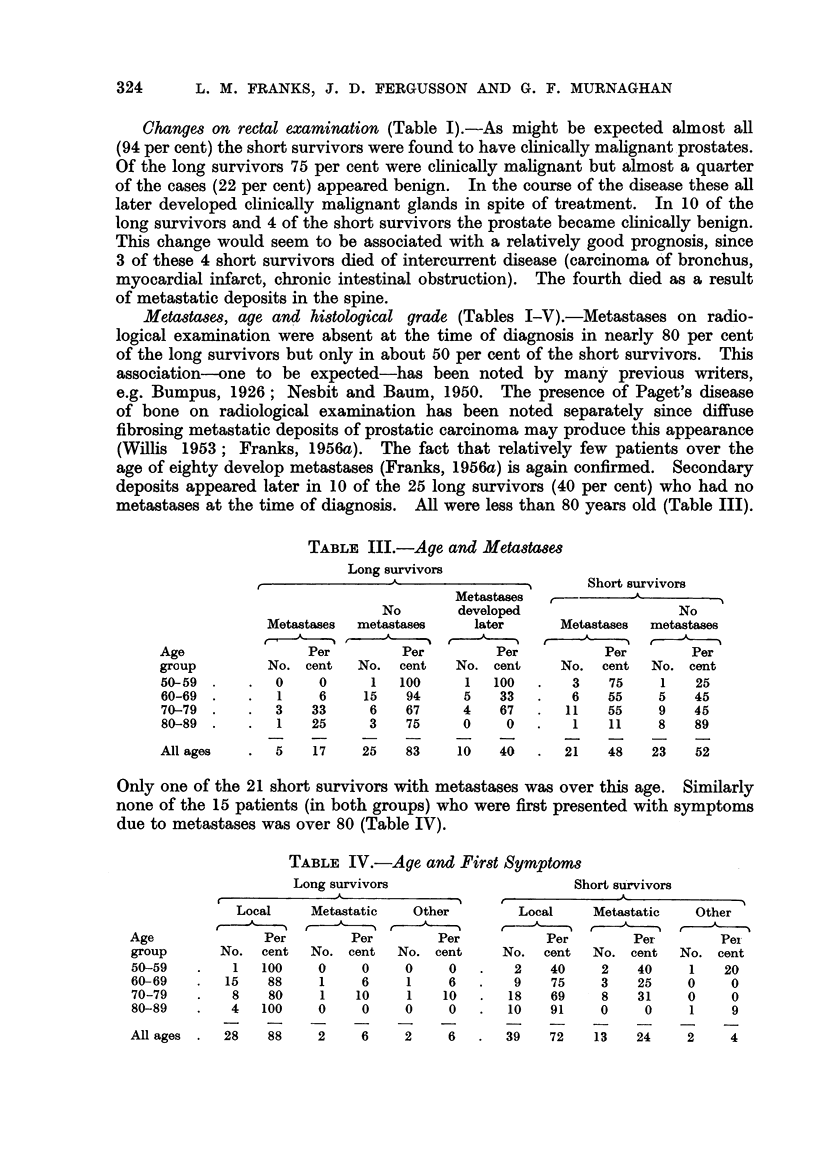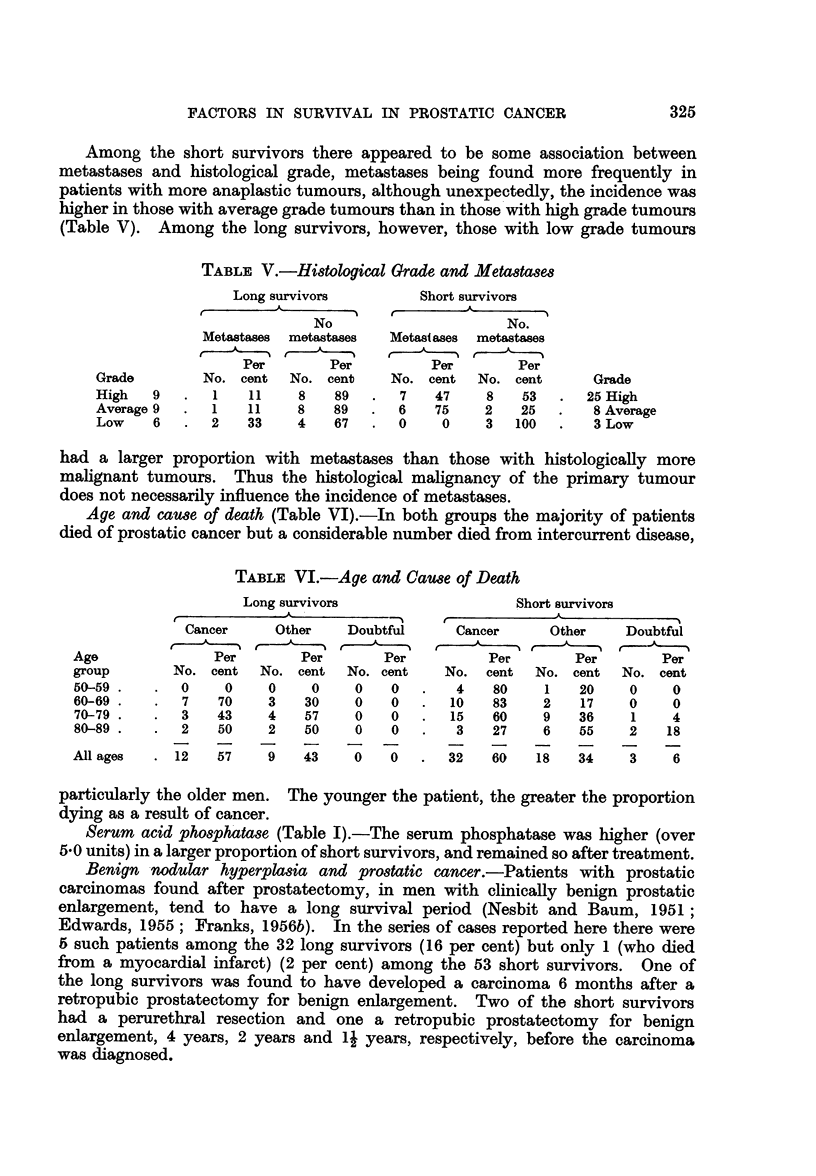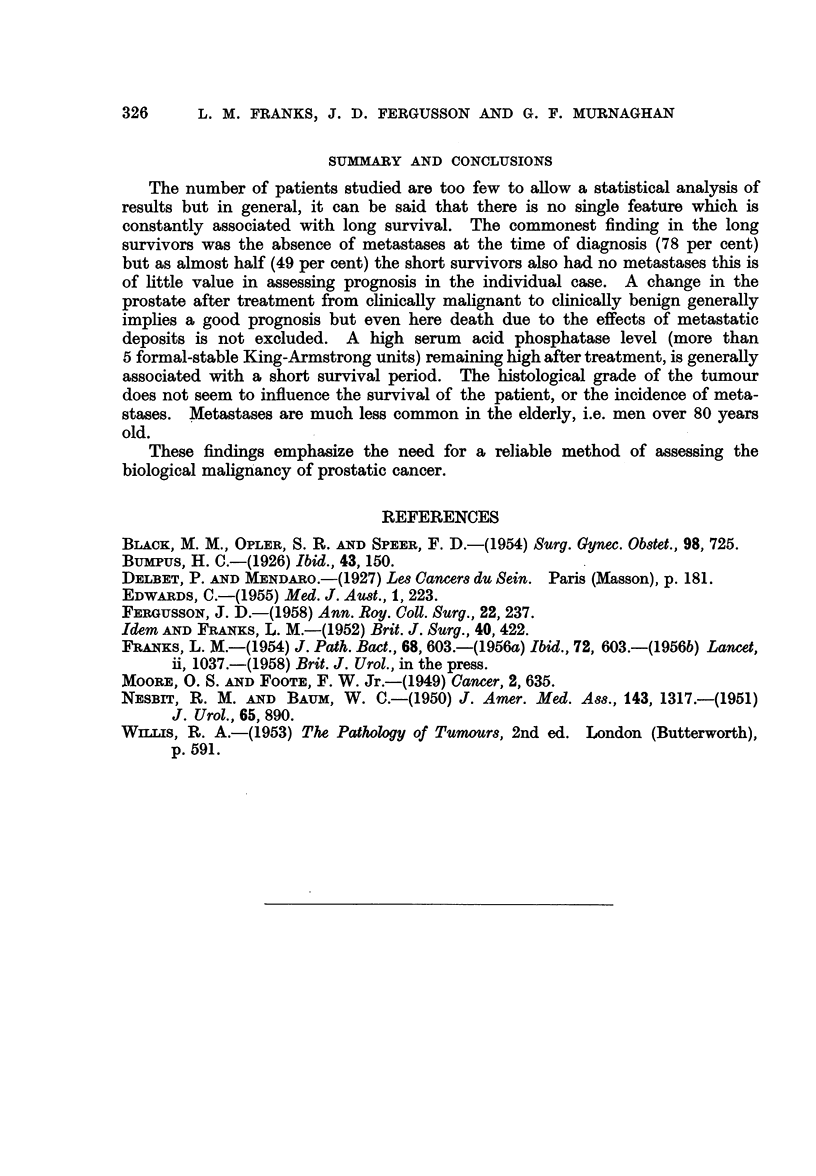# An Assessment of Factors Influencing Survival in Prostatic Cancer: The Absence of Reliable Prognostic Features

**DOI:** 10.1038/bjc.1958.38

**Published:** 1958-09

**Authors:** L. M. Franks, J. D. Fergusson, G. F. Murnaghan


### AN ASSESSMENT OF FACTORS INFLUENCING SURVIVAL IN PROSTATIC CANCER: THE ABSENCE OF RELIABLE PROGNOSTIC FEATURES

L. M. FRANKS, J. D. FERGUSSON AND G. F. MURNAGHAN*

*From the Imperial Cancer Research Fund Laboratories, Lincoln's Inn Fields, and the Institute of Urology (University of London), London, W.C.2*

Received for publication July 25, 1958

THE immediate and often dramatic improvement in patients with prostatic cancer, after endocrine treatment, tends to obscure the fact that a relatively small proportion of cases live for more than 5 years (see Franks, 1958, for review). The object of the present investigation was to see whether there were any specific features, using routine clinical and pathological methods, by which patients likely to live for more than 5 years could be recognised at the time of diagnosis.

### MATERIAL

There were 32 patients, surviving 5 years or more from the time of diagnosis, irrespective of the time of first appearance of symptoms, and in 24 of these cases histological sections from biopsies made at the time of diagnosis were available. This group was compared with another group from the same series of cases, living for less than 5 years. There were 53 patients in this latter group, and 26 of these had initial biopsy section available. The whole of this second group of " short survivors " had come to autopsy and the diagnosis and probable cause of death were confirmed histologically.

### METHODS

From haematoxylin and eosin stained paraffin sections of the initial biopsy material the tumours were graded, using a similar method to that used in an earlier investigation (Fergusson and Franks, 1952), and the presence or absence of basophil (" mucoid ") secretion in the tumour and of a surrounding stromal reaction, were recorded. The following clinical points were noted : first symptoms (urinary or due to metastatic disease), whether the prostate was clinically carcinomatous on rectal examination, the presence or absence of metastases on radiological examination when first seen, the formal-stable serum acid phosphatase when first seen, and the cause of death—whether due to prostatic cancer or intercurrent disease. Changes in the clinical state of the prostate, the serum acid phosphatase and the later development of metastases were also studied. The clinical changes in a larger unselected series of cases has been reported in detail by Fergusson (1958).

### RESULTS

The results are shown in detail in Tables I–VI and only the main points will be discussed below. The number of cases in each group and the percentage of

* Present address : Department of Surgical Science, University of Edinburgh, Edinburgh, 8.

TABLE I.—*Summary of Results*

| | Long survivors (5 years or more) | | | Short survivors (less than 5 years) | |
|---|---|---|---|---|---|
| | No. | Per cent of group | | No. | Per cent of group |
| *Age—* | | | | | |
| 50–59 | 1 | 3 | | 5 | 9 |
| 60–69 | 17 | 53 | | 12 | 23 |
| 70–79 | 10 | 31 | | 25 | 47 |
| 80–89 | 4 | 13 | | 11 | 21 |
| *Grade—* | | | | | |
| High | 9 | 37 | | 15 | 58 |
| Average | 9 | 37 | | 8 | 31 |
| Low | 6 | 25 | | 3 | 11 |
| Not graded | 8 | — | | 27 | — |
| *Stromal reaction—* | | | | | |
| Present | 8 | 38 | | 9 | 47 |
| Absent | 13 | 62 | | 10 | 53 |
| *Basophil secretion—* | | | | | |
| Present | 8 | 40 | | 5 | 26 |
| Absent | 12 | 60 | | 14 | 74 |
| *First symptoms—* | | | | | |
| Local | 28 | 88 | | 39* | 72 |
| Metastatic | 2 | 6 | | 13* | 24 |
| Incidental | 2 | 6 | | 2 | 4 |
| *Initial rectal examination—* | | | | | |
| Malignant (+) | 24 | 75 | | 50 | 94 |
| Benign (−) | 7 | 22 | | 3 | 6 |
| Doubtful (?) | 1 | 3 | | 0 | 0 |
| *Later rectal examination—* | | | | | |
| + → − | 10 | 31 | | 4 | 12 |
| − → + | 7 | 22 | | 1 | 3 |
| ? → + | 1 | 3 | | 0 | 0 |
| Unchanged | 14 | 44 | | 29 | 85 |
| *Metastases at diagnosis—* | | | | | |
| Present | 5 | 16 | | 21 | 45 |
| Paget's disease | 2 | 6 | | 3 | 6 |
| Absent | 25 | 78 | | 23 | 49 |
| *Metastases later—* | | | | | |
| Present | 15 | 47 | | 22 | 76 |
| Paget's disease | 2 | 6 | | 1 | 3 |
| Absent | 15 | 47 | | 6 | 21 |
| *Serum acid phosphatase. Initial—* | | | | | |
| 0 –1·9 K.A. units (Formal-stable) | 13 | 45 | | 19 | 43 |
| 2·0–4·9 | 13 | 45 | | 11 | 25 |
| 5·0– or more | 3 | 10 | | 14 | 32 |
| *Serum acid phosphatase. Later—* | | | | | |
| 0 –1·9 | 25 | 83 | | 23 | 60 |
| 2·0–4·9 | 5 | 17 | | 9 | 24 |
| 5·0 or more | 0 | 0 | | 6 | 16 |
| *Cause of death—* | | | | | |
| Cancer | 12 | 57 | | 32 | 64 |
| Other | 9 | 43 | | 18 | 36 |
| *Alive or cause of death doubtful* | 11 | — | | 3 | — |

* One patient had symptoms of both local and metastatic disease.

the group is given but, as in general the numbers are small, the percentages can only be regarded as a guide for ease of comparison. The apparent discrepancies in numbers are due to the fact that all investigations were not carried out in a few cases.

*Age* (Table I).—The age incidence in both long and short survivors follows the same general pattern in that most of the cases are between 60 and 80 years of age. Only one of the six patients between 50 and 60 years lived for more than 5 years. Apart from this the differences in age distribution in the two groups are probably not significant.

*Histological grade* (Tables I and II).—In both groups the number of patients with tumours of a low grade of malignancy is small, but there were more in the group of long survivors. Although rather more of the short survivors had high-grade tumours (58 per cent) nearly 40 per cent of the long survivors also had similar tumours. Thus the survival period is not directly related to the histological malignancy of the tumour. There appears to be no direct association between histological grade and age (Table II).

TABLE II.—*Age and Histological Grade*

| | Long survivors | | | | | | Short survivors | | | | | |
|---|---|---|---|---|---|---|---|---|---|---|---|---|
| | High | | Average | | Low | | High | | Average | | Low | |
| Age group | No. | Per cent | No. | Per cent | No. | Per cent | No. | Per cent | No. | Per cent | No. | Per cent |
| 50–59 | 0 | 0 | 0 | 0 | 0 | 0 | 4 | 100 | 0 | 0 | 0 | 0 |
| 60–69 | 5 | 36 | 7 | 50 | 2 | 14 | 2 | 33 | 4 | 67 | 0 | 0 |
| 70–79 | 3 | 37 | 2 | 25 | 3 | 37 | 4 | 40 | 4 | 40 | 2 | 20 |
| 80–89 | 1 | 50 | 0 | 0 | 1 | 50 | 5 | 83 | 0 | 0 | 1 | 17 |
| All ages | 9 | 37 | 9 | 37 | 6 | 25 | 15 | 58 | 8 | 31 | 3 | 11 |

*Stromal reaction to the tumour* (Table I).—Since it might be thought that a stromal reaction in the tumour may represent a response by the host to the tumour, the extent of any fibrous reaction was noted. There are no significant differences between the groups. It has been suggested that a lymphoid reaction around the tumour is associated with a long survival period in cancer of the breast (Moore and Foote, 1949) and stomach (Black, Opler and Speer, 1954). However, in these prostatic tumours a lymphoid reaction was seen only in association with areas of chronic inflammation, not obviously related to the tumour.

*Basophil secretion in the tumours* (Table I).—Many well differentiated prostatic tumours may secrete a basophil mucoid material (Franks, 1954). Since it has been reported by some workers, e.g. Delbet and Mendaro (1927) and others, that mucoid secretion in breast tumours was associated with a relatively good prognosis, this factor was also assessed. This type of secretion was present more frequently in the long survivors (40 per cent of cases as compared with 26 per cent), but this may only reflect the greater number of relatively well differentiated tumours (average and low grades combined) in this group. (62 per cent as compared with 42 per cent).

*First symptoms* (Table I).—In both groups the presenting symptoms were local (urinary) in the great majority but symptoms due to metastasis were commoner in the short survivors—24 per cent as compared with 6 per cent.

*Changes on rectal examination* (Table I).—As might be expected almost all (94 per cent) the short survivors were found to have clinically malignant prostates. Of the long survivors 75 per cent were clinically malignant but almost a quarter of the cases (22 per cent) appeared benign. In the course of the disease these all later developed clinically malignant glands in spite of treatment. In 10 of the long survivors and 4 of the short survivors the prostate became clinically benign. This change would seem to be associated with a relatively good prognosis, since 3 of these 4 short survivors died of intercurrent disease (carcinoma of bronchus, myocardial infarct, chronic intestinal obstruction). The fourth died as a result of metastatic deposits in the spine.

*Metastases, age and histological grade* (Tables I–V).—Metastases on radiological examination were absent at the time of diagnosis in nearly 80 per cent of the long survivors but only in about 50 per cent of the short survivors. This association—one to be expected—has been noted by many previous writers, e.g. Bumpus, 1926; Nesbit and Baum, 1950. The presence of Paget's disease of bone on radiological examination has been noted separately since diffuse fibrosing metastatic deposits of prostatic carcinoma may produce this appearance (Willis 1953; Franks, 1956*a*). The fact that relatively few patients over the age of eighty develop metastases (Franks, 1956*a*) is again confirmed. Secondary deposits appeared later in 10 of the 25 long survivors (40 per cent) who had no metastases at the time of diagnosis. All were less than 80 years old (Table III).

TABLE III.—*Age and Metastases*

| | Long survivors | | | | | | Short survivors | | | |
|---|---|---|---|---|---|---|---|---|---|---|
| | Metastases | | No metastases | | Metastases developed later | | Metastases | | No metastases | |
| Age group | No. | Per cent | No. | Per cent | No. | Per cent | No. | Per cent | No. | Per cent |
| 50–59 | 0 | 0 | 1 | 100 | 1 | 100 | 3 | 75 | 1 | 25 |
| 60–69 | 1 | 6 | 15 | 94 | 5 | 33 | 6 | 55 | 5 | 45 |
| 70–79 | 3 | 33 | 6 | 67 | 4 | 67 | 11 | 55 | 9 | 45 |
| 80–89 | 1 | 25 | 3 | 75 | 0 | 0 | 1 | 11 | 8 | 89 |
| All ages | 5 | 17 | 25 | 83 | 10 | 40 | 21 | 48 | 23 | 52 |

Only one of the 21 short survivors with metastases was over this age. Similarly none of the 15 patients (in both groups) who were first presented with symptoms due to metastases was over 80 (Table IV).

TABLE IV.—*Age and First Symptoms*

| | Long survivors | | | | | | Short survivors | | | | | |
|---|---|---|---|---|---|---|---|---|---|---|---|---|
| | Local | | Metastatic | | Other | | Local | | Metastatic | | Other | |
| Age group | No. | Per cent | No. | Per cent | No. | Per cent | No. | Per cent | No. | Per cent | No. | Per cent |
| 50–59 | 1 | 100 | 0 | 0 | 0 | 0 | 2 | 40 | 2 | 40 | 1 | 20 |
| 60–69 | 15 | 88 | 1 | 6 | 1 | 6 | 9 | 75 | 3 | 25 | 0 | 0 |
| 70–79 | 8 | 80 | 1 | 10 | 1 | 10 | 18 | 69 | 8 | 31 | 0 | 0 |
| 80–89 | 4 | 100 | 0 | 0 | 0 | 0 | 10 | 91 | 0 | 0 | 1 | 9 |
| All ages | 28 | 88 | 2 | 6 | 2 | 6 | 39 | 72 | 13 | 24 | 2 | 4 |

Among the short survivors there appeared to be some association between metastases and histological grade, metastases being found more frequently in patients with more anaplastic tumours, although unexpectedly, the incidence was higher in those with average grade tumours than in those with high grade tumours (Table V). Among the long survivors, however, those with low grade tumours

TABLE V.—*Histological Grade and Metastases*

| | Long survivors | | | | Short survivors | | | | |
|---|---|---|---|---|---|---|---|---|---|
| | Metastases | | No metastases | | Metastases | | No. metastases | | |
| Grade | No. | Per cent | No. | Per cent | No. | Per cent | No. | Per cent | Grade |
| High 9 | 1 | 11 | 8 | 89 | 7 | 47 | 8 | 53 | 25 High |
| Average 9 | 1 | 11 | 8 | 89 | 6 | 75 | 2 | 25 | 8 Average |
| Low 6 | 2 | 33 | 4 | 67 | 0 | 0 | 3 | 100 | 3 Low |

had a larger proportion with metastases than those with histologically more malignant tumours. Thus the histological malignancy of the primary tumour does not necessarily influence the incidence of metastases.

*Age and cause of death* (Table VI).—In both groups the majority of patients died of prostatic cancer but a considerable number died from intercurrent disease,

TABLE VI.—*Age and Cause of Death*

| | Long survivors | | | | | | Short survivors | | | | | |
|---|---|---|---|---|---|---|---|---|---|---|---|---|
| | Cancer | | Other | | Doubtful | | Cancer | | Other | | Doubtful | |
| Age group | No. | Per cent | No. | Per cent | No. | Per cent | No. | Per cent | No. | Per cent | No. | Per cent |
| 50–59 | 0 | 0 | 0 | 0 | 0 | 0 | 4 | 80 | 1 | 20 | 0 | 0 |
| 60–69 | 7 | 70 | 3 | 30 | 0 | 0 | 10 | 83 | 2 | 17 | 0 | 0 |
| 70–79 | 3 | 43 | 4 | 57 | 0 | 0 | 15 | 60 | 9 | 36 | 1 | 4 |
| 80–89 | 2 | 50 | 2 | 50 | 0 | 0 | 3 | 27 | 6 | 55 | 2 | 18 |
| All ages | 12 | 57 | 9 | 43 | 0 | 0 | 32 | 60 | 18 | 34 | 3 | 6 |

particularly the older men. The younger the patient, the greater the proportion dying as a result of cancer.

*Serum acid phosphatase* (Table I).—The serum phosphatase was higher (over 5·0 units) in a larger proportion of short survivors, and remained so after treatment.

*Benign nodular hyperplasia and prostatic cancer.*—Patients with prostatic carcinomas found after prostatectomy, in men with clinically benign prostatic enlargement, tend to have a long survival period (Nesbit and Baum, 1951; Edwards, 1955; Franks, 1956*b*). In the series of cases reported here there were 5 such patients among the 32 long survivors (16 per cent) but only 1 (who died from a myocardial infarct) (2 per cent) among the 53 short survivors. One of the long survivors was found to have developed a carcinoma 6 months after a retropubic prostatectomy for benign enlargement. Two of the short survivors had a perurethral resection and one a retropubic prostatectomy for benign enlargement, 4 years, 2 years and 1½ years, respectively, before the carcinoma was diagnosed.

### SUMMARY AND CONCLUSIONS

The number of patients studied are too few to allow a statistical analysis of results but in general, it can be said that there is no single feature which is constantly associated with long survival. The commonest finding in the long survivors was the absence of metastases at the time of diagnosis (78 per cent) but as almost half (49 per cent) the short survivors also had no metastases this is of little value in assessing prognosis in the individual case. A change in the prostate after treatment from clinically malignant to clinically benign generally implies a good prognosis but even here death due to the effects of metastatic deposits is not excluded. A high serum acid phosphatase level (more than 5 formal-stable King-Armstrong units) remaining high after treatment, is generally associated with a short survival period. The histological grade of the tumour does not seem to influence the survival of the patient, or the incidence of metastases. Metastases are much less common in the elderly, i.e. men over 80 years old.

These findings emphasize the need for a reliable method of assessing the biological malignancy of prostatic cancer.

### REFERENCES

BLACK, M. M., OPLER, S. R. AND SPEER, F. D.—(1954) *Surg. Gynec. Obstet.*, **98**, 725.
BUMPUS, H. C.—(1926) *Ibid.*, **43**, 150.
DELBET, P. AND MENDARO.—(1927) *Les Cancers du Sein.* Paris (Masson), p. 181.
EDWARDS, C.—(1955) *Med. J. Aust.*, **1**, 223.
FERGUSSON, J. D.—(1958) *Ann. Roy. Coll. Surg.*, **22**, 237.
*Idem* AND FRANKS, L. M.—(1952) *Brit. J. Surg.*, **40**, 422.
FRANKS, L. M.—(1954) *J. Path. Bact.*, **68**, 603.—(1956*a*) *Ibid.*, **72**, 603.—(1956*b*) *Lancet*, ii, 1037.—(1958) *Brit. J. Urol.*, in the press.
MOORE, O. S. AND FOOTE, F. W. JR.—(1949) *Cancer*, **2**, 635.
NESBIT, R. M. AND BAUM, W. C.—(1950) *J. Amer. Med. Ass.*, **143**, 1317.—(1951) *J. Urol.*, **65**, 890.
WILLIS, R. A.—(1953) *The Pathology of Tumours*, 2nd ed. London (Butterworth), p. 591.